# Management of liver trauma in adults

**DOI:** 10.4103/0974-2700.76846

**Published:** 2011

**Authors:** Nasim Ahmed, Jerome J Vernick

**Affiliations:** Department of Surgery & Division of Trauma and Surgical Critical Care, Jersey Shore University Medical Center 1945 State Rt. 33, Neptune, US

**Keywords:** Liver, injury, damage control surgery

## Abstract

The liver is one of the most commonly injured organs in abdominal trauma. Recent advancements in imaging studies and enhanced critical care monitoring strategies have shifted the paradigm for the management of liver injuries. Nonoperative management of both low- and high-grade injuries can be successful in hemodynamically stable patients. Direct suture ligation of bleeding parenchymal vessels, total vascular isolation with repair of venous injuries, and the advent of damage control surgery have all improved outcomes in the hemodynamically unstable patient population. Anatomical resection of the liver and use of atriocaval shunt are rarely indicated.

## INTRODUCTION

The liver is one of the most frequently injured organs in abdominal trauma.[1,2] The anterior location in the abdominal cavity and fragile parenchyma with easily disrupted Glisson’s capsule make this organ vulnerable to injury.

There is a paradigm shift in the management of liver trauma due to advancements of diagnostic and therapeutic modalities. About a century ago, Pringle conducted an animal experiment, occluding the porta hepatis in liver trauma while repairing the injuries.[3] However, application of the same principle in trauma victims led to high mortality.[4] Since 1965, the introduction of diagnostic peritoneal lavage (DPL) has led to many nontherapeutic laparotomies in previously unsuspected low-grade injuries.[5] Operative intervention in high-grade injuries may result in high mortality as well.[4,6] Introduction of computed tomography (CT) scan, use of ultrasonography in trauma, availability of angiography, enhanced critical care monitoring and damage control surgery have revolutionized the management of liver trauma. Numerous studies have shown better outcome with conservative management.[7,8] Though there is a broader consensus regarding the nonoperative approach even in high-grade injuries, however, some controversies still exist.[8–18]

This review discusses the diagnostic modality and therapeutic approach to liver trauma.

## CLASSIFICATION OF THE LIVER INJURIES

Liver injury is classified based on severity of the injury [Table 1].[19]

**Table 1:** Grading of liver injury based on American Association of Surgery for trauma (AAST)[19]

Grade	Type	Injury description
I	Hematoma	Subcapsular, nonexpanding, <10 cm surface area
	Laceration	Capsular tear, nonbleeding, <1 cm parenchymal depth
II	Hematoma	Subcapsular, nonexpanding, 10–50% surface area; intraparenchymal nonexpanding <10 cm diameter
	Laceration	Capsular tear, active bleeding, 1–3 cm parenchymal depth <10 cm in length
III	Hematoma	Subcapsular, >50% surface area or expanding; ruptured subcapsular hematoma with active bleeding; intraparenchymal hematoma >10 cm or expanding
	Laceration	>3 cm parenchymal depth
IV	Hematoma	Ruptured intraparenchymal hematoma with active bleeding
	Laceration	Parenchymal disruption involving 25–75% of hepatic lobe or one to three Couinaud’s segments within a single lobe
V	Laceration	Parenchymal disruption involving >75% of hepatic lobe or >3 Couinaud’s segments within a single lobe
	Vascular	Juxtahepatic venous injuries (i.e., retrohepatic vena cava/central major hepatic veins)
VI	Vascular	Hepatic avulsion

## DIAGNOSIS

Imaging studies are the main diagnostic modality of evaluation of presence or absence of liver trauma.

### Ultrasonography

Ultrasonography is a noninvasive procedure and highly operator-dependent. Focused assessment by ultrasound for trauma (FAST) has been advocated in initial trauma evaluation.[20] The purpose of this exam is to provide a quick bedside assessment for hemoperitoneum and hemopericardium. A FAST exam consists of sonographic evaluation of pericardium, right upper quadrant, including Morrison’s pouch, left upper quadrant and the pelvis. This evaluation is not designed to identify the degree of organ injuries, but rather the presence of blood. The sensitivity and specificity of this examination are 63–100% and 95–100%, respectively.[20–22] Negative FAST examination does not exclude intra-abdominal injuries or hemoperitonium. Retroperitonial injuries and hollow viscus injuries can also be missed by ultrasound evaluation.

Recent advancement of contrast-enhanced sonography improved the diagnostic accuracy in terms of conspicuity, size and completeness of the injury, as compared to non-contrast sonography. It is also similar to CT scan in terms of identification of ongoing hemorrhage in the liver.[23]

### Computed tomography scan

CT scan is the first imaging study which gives relatively detailed delineation of solid organ injuries and retroperitoneal injuries as well. CT scan is the standard imaging study for hemodynamically stable patients following blunt trauma.[24,25] Severity of injuries is also graded based on CT scan examination.[19] Extravasation of contrast demonstrated on CT scan (35–40 HU) indicates active bleeding from the injury site and further intervention is needed.[26,27]

The sensitivity and specificity of the CT scan for liver injuries are 92–97% and 98.7%, respectively.[28]

CT scan plays an integral role in the nonoperative management of liver injuries. Follow-up CT scan is recommended for high-grade injuries (grades IV–V) in 7–10 days to determine the injury status and complications as well.[8,29] CT scan-guided percutaneous drainage may also be performed when complications such as biloma and intra-abdominal collections occur.

### Angiogram and angioembolization

Angiography plays a vital role in the conservative management of the liver injury. Extravasation of contrast seen on CT scan requires emergency angiography and angioembolization in hemodynamically stable patients. Post-operative angioembolization is also reported in damage control surgery prior to removal of packing, if rebleeding is suspected.[30,31] The sensitivity and specificity of angiogram identifying active bleeding in liver injuries is 75% and the success rate of controlling the hemorrhage is 68–93%.[11,30,32] The multidisciplinary approach to conservative management of high-grade liver injuries shows better outcome with less blood transfusion, early recovery time and less intensive care days. The mortality is low as well.[33]

### Diagnostic peritoneal lavage

DPL was one of the most common modalities used in the diagnostic evaluation for blunt abdominal trauma in the mid-20^th^ century.[29] This procedure is very sensitive for hemoperitoneum. Positive DPL led to a rate of almost 30% non-therapeutic, unnecessary laparotomies.[5,34] Widely available CT scans and the introduction of FAST have generally replaced the invasive DPL. However, the Advanced Trauma Life Support course (ATLS) still includes this modality and it remains one of the skills that physicians need to learn for ATLS certification.

## MANAGEMENT

Management of liver injury has evolved in the last 25 years. Advancement of imaging studies plays a key role in the conservative approach. In early 1970, more than 80% of the liver injuries were managed operatively. In late 1990, 80–90% of these injuries were successfully managed by nonoperative means.

### Nonoperative management

#### Penetrating injury

Nonoperative management is now recommended for stab wound as well as low-velocity gunshot wound to right upper quadrant in stable patients, if other injuries have been excluded which require laparotomy.[35,36] Most of the injuries which fall in this category are grade I and grade II injuries.

#### Blunt injury

In blunt liver trauma, nonoperative management is a standard of care in hemodynamically stable patients. It is not the grade of the injury, but rather the hemodynamic parameters of the patient which dictate the conservative versus operative management decision. The patient’s positive response to an initial fluid bolus or maintenance of a stable hemodynamic state allows for a CT scan of abdomen and pelvis. If extravasation is identified, angiogram and angioembolization should be considered. Failures of these steps then mandate operative intervention.

The most common reasons for failure are advanced age, delayed bleeding, hypotension and active extravasation of contrast not controlled by angioembolization.[33,37–39]

There is an overall survival benefit and 23% reduction of mortality for conservative approach in blunt liver injury.[40–42]

### Operative management

#### Penetrating injury

Recent literature supports operative intervention only in hemodynamically unstable patients, usually as a result of a high-velocity gunshot wound. Other indication for operative intervention is an associated hollow viscus injury.[43]

Trunkey has described the operative procedure for unstable gunshot wounds to the liver.[29] If the patient is unstable or deteriorating in the emergency room, patients should be taken to the operating room within 15 minutes. Activation of massive blood transfusion protocol, four quadrant packing, direct compression and rapid control of fecal contamination are the initial steps. Debridement, ligation of the bleeding vessel, lobectomy and repair of venous injury under total vascular isolation are the best strategies with good outcome. If the triad of coagulopathy, acidosis and hypothermia are encountered during this phase of the repair, perihepatic packing and temporary closure of the abdominal incision with transfer to intensive care unit (ICU) should be the priority. The patient should be taken back to operating room as soon as the metabolic derangement is corrected and rewarming has occurred.

#### Blunt injury

The main indication of the operative approach to the blunt liver injury is hemodynamic instability, not the grading of the injury. Although a higher grade injury has higher potential for failure of nonoperative management, hemodynamic instability remains the most important branch of the decision tree indicating operative intervention.

Rebleeding, constant decline of hemoglobin and increased transfusion requirement, as well as the failure of angioembolization of actively bleeding vessels are a few factors which indicate the need forlaparotomy.[33,37–39]

The operative approach has also evolved over the last two decades. Direct suture ligation of the parenchymal bleeding vessel, perihepatic packing, repair of venous injury under total vascular isolation and damage control surgery with utilization of preoperative and/or postoperative angioembolization are the preferred methods, compared to anatomical resection of the liver and use of the atriocaval shunt.[9,44–51]

### Operative procedure for liver injuries

The first and the most important step in operative management of blunt liver injury is to pack all four quadrants with laparotomy pads and manually compress the liver using both hands for 15–20 minutes. This allows the anesthesiologist to catch up with the resuscitation. Then remove the lower quadrant packing first, followed by left upper quadrant and finally right upper quadrant. If the spleen is actively bleeding, splenectomy should be performed. Assess the liver laceration and identify the bleeding vessel. Direct suture ligation should be performed using 3-0 or 4-0 absorbable suture. A patch of omentum can be used to fill the gap created by the laceration. If bleeding continues, then perform the Pringle maneuver (apply a noncrushing clamp through the foramen of Winslow).[3] The clamp can be safely applied up to 1 hour.

### Operative approach for hepatic vein and/or retrohepatic caval injuries

If bleeding continues despite the Pringle maneuver, then retrohepatic, caval or hepatic vein injury should be suspected.

The preferred method for caval and hepatic vein injury is total vascular isolation.[47] The procedure consists of performing a Pringle maneuver, and clamping of the inferior vena cava above and below the injury. Superiorly, the inferior vena cava can be isolated just below the diaphragm or through the pericardium by extending the incision to a median sternotomy and inferiorly, just above the renal veins. This approach allows direct repair of the vascular injury. Aortic clamping is not recommended for the vena caval or hepatic vein injury.[29] The vascular isolation technique has reported a better survival rate compared to atriocaval shunt.[47,52] Anatomical lobectomy is rarely performed; however, in the hands of an expert, the outcome is very good.[53,54]

During the operative repair, if the patient develops coagulopathy, acidosis, or hypothermia, damage control surgery should be considered.

### Damage control surgery

Damage control surgery includes perihepatic packing and closure of the abdominal incision either using a Bogata bag or partial closure of proximal abdominal incision. Kreig *et al*. recommend six folded laparatomy pads to be placed between the liver and the abdominal wall to obtain tamponade.[44] The patient should be transferred to the ICU as soon as possible for continued resuscitation and warming. As soon as the metabolic derangement is corrected, the patient should be taken back to operating room for re-exploration. The timing of re-exploration depends upon the correction of acidosis, coagulopathy and hypothermia. Usually, 12–24 hours is the safe period for re-exploration and formal completion of the surgery.

### Role of hemostatic agents in liver trauma

A number of commercial hemostatic agents are readily available and can be used as an adjunct after repair of liver injuries. The most commonly used agents are gelatin gelfoams, oxidized cellulose, microfibrillar collagen, thrombin, thrombin with gelatin (floseal) and fibrin sealant (tisseel).[55]

### Application of extracorporeal circulation in the massive liver and/or retrohepatic caval injury

The use of extracorporeal circulation devices during the repair of the juxtahepatic caval injuries has been noted in the past with variable success. The concept behind this device is to bypass the flow from the injured area using an extracorporeal circuit, with or without an active pump. Therefore, repair can be performed in a bloodless field. These devices increase the complexity of the operation, and the physician must be familiar with the technique and concept as well. Successful use of venovenous bypass following clamping of the inferior vena cava during anhepatic phase of liver transplant provided the idea in the management of retrohepatic caval injuries.[56] This technique allows blood to be diverted from the inferior vena cava, with or without portal vein decompression, and drain it into the right atrium either directly or through internal jugular vein or superior vena cava.[57–60]

### Liver transplantation in massive liver and hepatic venous and retrohepatic caval injury

Orthotopic liver transplantation has been reported as an extreme measure in massive hepatic venous and retrohepatic caval injuries.[61–65] Since the mortality rate associated with these injuries is extremely high and there is a shortage of organs available for procurement as well, the indication for liver transplantation is very restricted. Most of the indications described in the literature are uncontrolled bleeding despite repeated previous surgery and acute or progressive liver failure following repair of injury. In the last two decades, less than 20 liver transplantations have been performed for liver trauma with variable success, however, the biggest series was reported by Delis and colleagues, in which three out of four patients survived after liver transplantation.[66] A case of extracorporeal repair and “autotransplantation” has also been reported in the case of total avulsion of hepatic veins and a retrohepatic caval injury.[67] This patient’s first operation consisted of the ligation of hepatic veins and inferior retrohepatic vena cava, and then transfer to another facility where the second operation was performed. Immediately upon arrival, extracorporeal bypass was established between left femoral vein and left axillary vein, using a centrifugal pump. Additional cannula was inserted into the inferior mesenteric vein threaded back to the portal vein and connected to the bypass circulation. After cross-clamping the hepatoduodenal ligament, the liver was removed *en bloc* with the retrohepatic vena cava. After *ex vivo* repair of the massive liver injury was completed, the liver was re-transplanted. Revascularization was done just over 3 hours without using any extension grafts for the portal vein or hepatic artery. After establishing adequate bleeding control, the procedure was completed with an end-to-end choledochocholedochostomy over a T-tube.

### Role of interventional radiology in liver injury

The interventional radiologist plays an integral role in the nonoperative management of liver injuries. Angiography and angioembolization has become the gold standard in the management of liver injuries for hemodynamically stable patients, if a contrast extravasation is seen on CT scan. Furthermore, conservative management may cause vascular/or biliary complications, particularly in high-grade injuries which require imaging intervention. Post-traumatic pseudoaneurysm, intrahepatic arteriovenous fistula and hemobilia are a few vascular complications which may appear following liver injuries and angioembolization is the first step in the management of these complications.[68] Symptomatic biloma, liver and intra-abdominal abscesses can also be successfully managed by CT-guided percutaneous drainage.[69]

## CONCLUSION

Management of liver injury has evolved over the last two decades. Hemodynamic status, not the grade of the injury, should dictate the management. CT scan of the abdomen and pelvis is a standard diagnostic modality in hemodynamically stable patients. Extravasation of contrast during CT scans requires further intervention. Unstable patients should mandate emergency laparotomy. Direct control of bleeding vessels, vascular isolation and damage control surgery are preferred and the most popular approaches compare to anatomical resection of liver and the use of an aortocaval shunt.
